# A critical review of traditional medicine and traditional healer use for malaria and among people in malaria-endemic areas: contemporary research in low to middle-income Asia-Pacific countries

**DOI:** 10.1186/s12936-015-0593-7

**Published:** 2015-03-01

**Authors:** Dwi L Suswardany, David W Sibbritt, Sudibyo Supardi, Sungwon Chang, Jon Adams

**Affiliations:** Australian Research Centre in Complementary and Integrative Medicine (ARCCIM), Faculty of Health, University of Technology Sydney, Level 7, Building 10, 235-253 Jones St, Broadway, Sydney, NSW 2007 Australia; Universitas Muhammadiyah Surakarta, Surakarta, Central Java Indonesia; National Institute of Health Research and Development, Ministry of Health Indonesia, Jakarta, Indonesia

**Keywords:** Malaria, Traditional medicine, Traditional healer

## Abstract

**Background:**

Malaria is a leading health threat for low to middle-income countries and around 1.8 billion people in the Southeast Asian region and 870 million people in the Western Pacific region remain at risk of contracting malaria. Traditional medicine/traditional healer (TM/TH) use is prominent amongst populations in low- to middle-income countries and constitutes an important issue influencing and potentially challenging effective, safe and coordinated prevention and treatment strategies around malaria. This paper presents the first critical review of literature on the use of TM/TH for malaria prevention and treatment in low- to middle-income countries in the Asia-Pacific region.

**Methods:**

A comprehensive search of English language, peer-reviewed literature reporting TM and/or TH use for malaria or among people in malaria-endemic areas in low- to middle-income Asia-Pacific countries published between 2003 and 2014 was undertaken.

**Results:**

Twenty-eight papers reporting 27 studies met the inclusion criteria. Prevalence of TM/TH use for malaria treatment ranged from 1 to 40.1%. A majority of studies conducted in rural/remote areas reported higher prevalence of TM/TH use than those conducted in mixed areas of urban, semi-urban, rural, and remote areas. Those utilizing TM/TH for malaria are more likely to be: women, people with lower educational attainment, people with lower household income, those with farming occupations, and those from ethnic minorities (identified from only three studies). The majority of adult participants delayed seeking treatment from a health centre or conventional providers while initially practicing TH use. The most common reasons for TM/TH use for malaria across the Asia-Pacific region are a lack of accessibility to conventional health services (due to geographical and financial barriers), faith in traditional treatment, and the perception of lower severity of malaria symptoms.

**Conclusion:**

This review has provided crucial insights into the prevalence and profile of TM/TH use for malaria. Those managing and providing conventional programmes, treatment and care for malaria in the Asia-Pacific should remain mindful of the possible use of TM/TH amongst community members and patients.

**Electronic supplementary material:**

The online version of this article (doi:10.1186/s12936-015-0593-7) contains supplementary material, which is available to authorized users.

## Background

Malaria remains a leading health threat for low- to middle-income country populations. While malaria control efforts over the last decade have decreased the incidence of malaria cases in more than half of the endemic countries in the Western Pacific and Southeast Asian regions [1], in more densely populated countries such as Bangladesh, India, Indonesia, and Myanmar, attempts to defeat malaria remain less successful [2]. Around 1.8 billion people in the Southeast Asian region and 870 million people in the Western Pacific region remain at risk of contracting malaria [1].

As a preventable and curable disease, most deaths and severe conditions resulting from malaria are avoidable through timely and effective treatment [3,4]. Timely initiation of diagnosis and treatment with recommended adequate drugs has the potential to produce low mortality rates, less advanced disease and to help eradicate malaria transmission amongst the community [5]. However, challenges facing the elimination of malaria still exist due to identified economic [6-8], geographical [9,10] and health system factors [4,11].

Access to health services in low- to middle-income countries is often limited (especially in rural and remote regions) [12,13]. Traditional medicine (TM) and traditional healers (TH) – those health care practices, treatments and providers that are indigenous to the culture and which have historically operated predominantly outside the state-funded healthcare system as well as beyond the practices and curriculum of the dominant medical profession [14] – are an important, popular component of health seeking and treatment for many people in low to middle-income countries in Asia-Pacific, as elsewhere [15]. TM/TH is often utilized by the general population on a regular basis for maintaining health [16,17] and/or for both chronic and acute diseases [18-20]. The vast majority of TM/TH use is prior to or in the absence of conventional medical services [21,22]. One study has shown TM/TH users are likely to be women, university graduates or low-income respondents [23]. In contrast, other studies have found men more likely to use TM/TH than women [24,25] or have identified no relationship between age, education, and income and TM use [18].

Given the significant prevalent rates of TM/TH use in low- to middle-income countries [26] and that TM/TH use in many cases is the only healthcare available/accessible to many people in these communities [27,28], TM/TH use is an important issue challenging and/or influencing the effective, safe and coordinated provision of conventional medical services to these populations [29], a situation affecting the prevention and treatment strategies around a range of health conditions and diseases, including malaria. As such, it is imperative that those providing, managing and developing preventive strategies and policies and conventional healthcare services with regard to malaria are cognizant of the behaviour, decision-making and perceptions of community members relating to TM/TH use.

This paper presents the first critical review of literature on the use of TM/TH with regard to malaria prevention and treatment in low- to middle-income countries in the Asia-Pacific region. The review synthesizes empirical work and findings providing a critical appraisal of study methodology and design as well as highlighting important gaps in understanding the use of TM/TH by malaria patients and or people in malaria-endemic areas in low- to middle-income countries across the Asia-Pacific to help guide future research on this topic.

## Methods

### Research design

The review examines the current prevalence and profile of TM/TH use among people with malaria and/or among people in malaria-endemic areas in low- to middle-income countries in the Asia-Pacific region. The inclusion of specific countries in the region that meet such criteria is based upon those identified by the World Bank [30]. A comprehensive search of literature published between January 2003 and October 2014 was undertaken. Seven databases were utilized in the review: Academic Search Complete (Ebsco), CINAHL, MEDLINE (Ovid), Proquest, Scopus, and AMED. In addition, Google Scholar was employed to identify any further relevant literature. All are well-known, mainstream databases of health and medical scholarship and collectively provide excellent access to a range of disciplinary works appropriate to the review focus. The search strategy can be found in the Table 1.

**Table 1:** **Literature review search strategy**

**Databases**	1. Academic Search Complete (Ebsco)
	2. CINAHL
	3. MEDLINE (Ovid)
	4. Proquest
	5. Scopus
	6. AMED
**Key/Search terms**	Malaria or febrile or fever or ‘mosquito-borne illness’ or ‘mosquito-borne disease’ or ‘mosquito-borne infectious illness’ or ‘mosquito-borne infectious disease’ or ‘mosquito borne illness’ or ‘mosquito borne disease’ or ‘mosquito borne infectious illness’ or ‘mosquito borne infectious disease’and combine them all with ‘treatment-seeking’ or ‘health-seeking’ or ‘care-seeking’ or ‘treatment seeking’ or ‘health seeking’ or ‘care seeking’ or ‘traditional medicine’ or ‘traditional healer’ or ‘traditional therapy’ or ‘traditional health care’ or ‘traditional healthcare’ or ‘traditional treatment’ or ‘Indigenous medicine’ or ‘indigenous healer’ or ‘indigenous therapy’ or ‘indigenous health care’ or ‘indigenous healthcare’ or ‘indigenous treatment’ or ‘traditional Chinese medicine’ or ‘traditional Chinese healer’ or ‘traditional Chinese therapy’ or ‘traditional Chinese health care’ or ‘traditional Chinese healthcare’ or ‘traditional Chinese treatment’ or Jamu or ‘herb’ or ‘medicinal plant’ or acupuncture or ‘Ayurveda/ayurvedic’ or ‘unani’ or ‘herbal oil’ or ‘faith healer’ or ‘mosquito repellent’
**Limits**	
**Month/year published**	January 2003-October 2014
**Language**	English
**Types of population**	Humans of all ages (people with malaria or people living in malaria-endemic areas)
**Location**	Low- to middle-income countries in the Asia-Pacific region
**Type of publication**	Peer-reviewed article
**Inclusion/exclusion criteria**	
**Types of studies**	Any studies reporting empirical research findings on treatment or prevention of malaria using traditional medicine or traditional healers
**Exclusion criteria**	editorials, correspondence, commentaries, case reports, clinical studies (including those utilizing randomized controlled trial designs), and papers not adopting systematic research design or data reporting procedures.

Search results were imported into EndNote X7 with duplicated items removed. The search was limited to those peer-reviewed articles published in English and reporting studies conducted in low- to middle-income Asia-Pacific countries. All retrieved titles and abstracts were screened to identify papers reporting empirical research findings. Papers identified as editorials, correspondence, commentaries, case reports, and writing that had not adopted systematic research design or data-reporting procedures were excluded. In line with the aim of the review, any papers reporting clinical studies (including those utilizing randomized controlled trial designs) were also excluded. If initial viewing of an abstract of a paper did not provide sufficient information to make an informed decision, the full article was located and examined by the authors prior to making a judgment regarding inclusion in the review. Relevant works were also searched by examining bibliographies of publications already identified.

### Search outcomes

**Figure 1:**
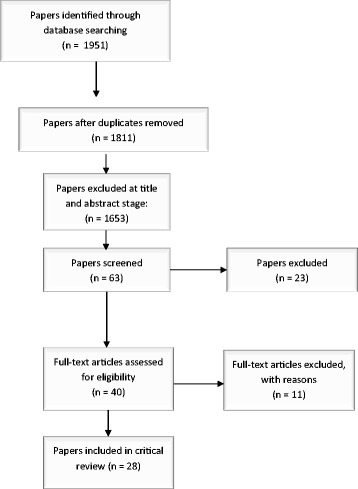
**Flow chart of included and excluded studies.**

The initial search identified 1,811 papers after duplicates were removed. Of these 1,811 papers, a total of 28 articles (reporting on 27 studies) identified during full text assessment met the inclusion criteria and were included in the review. Figure 1 reports the literature search process and Additional files 1 and 2 summarize the basic details of the included papers.

### Quality appraisal

In order to appraise the quality of the papers included in the review, a modified quality scoring system (Table 2), previously used for assessing complementary and alternative medicine (CAM) prevalence studies [31-33], was employed. These quality criteria reflect a combination of aspects of methodology, participants’ characteristics and TM/TH usage. Two authors assigned scores to the papers separately; the results were then compared and differences resolved by discussion. Table 3 reports the summary of total quality score of each paper. The details of the quality scores can be found in Additional file 3.

**Table 2:** **Description of quality scoring system for quantitative studies on traditional medicine/traditional healer use for malaria survey reviewed** [31-33]

**Dimensions of quality assessment**	**Codes**	**Points awarded**
*Methodology:*		
Representative sampling strategy	A	1
Sample size >500	B	1
Response rate >75%	C	1
Low recall bias (prospective data collection or retrospective data collection within past 12 months)	D	1
Confirmed malaria patients by health staff (microscopic test)	E	1
*Reporting of participants’ characteristics:*		
Status of malaria	F	1
Age	G	1
Indicator of socio-economic status (e.g., income, education)	H	1
Types of areas (urban/rural/remote)	I	1
*Reporting of traditional medicine/therapy use:*		
Definition of TM or modalities provided to respondents	J	1
Participants can name TM type/therapy/ modalities	K	1
Types of areas (urban/rural/remote)	L	1
TOTAL SCORES		12

**Table 3:** **Quality scoring summary of quantitative studies examining traditional medicine/traditional healer among populations in low- to middle-income countries in the Asia-Pacific region**

	Dimension of quality assessment*			
**Authors/year/country**	**Methodology**	**Reporting of participants’ characteristics**	**Reporting of TM/therapy use**	**Total score**
Al-Adhroey *et al.* [52], Malaysia	2 (CD)	4 (FGHI)	1 (K)	7
Al-Adhroey *et al*. [44], Malaysia	1 (D)	2 (FI)	1 (K)	4
Al-Taiar *et al.* [42], Yemen	3 (BDE)	4 (FGHI)	0	7
Bell *et al.* [45], Philippines	3 (CDE)	4 (FGHI)	1 (K)	8
Borah *et al*. [46], India	1 (E)	3 (FGH)	0	4
Chaturvedi *et al.* [35], India	3 (BCD)	4 (FGHI)	1 (J)	8
Das and Ravindran [47], India	2 (CD)	4 (FGHI)	0	6
Davy *et al.* [53], PNG	3 (BCD)	4 (FGHI)	0	7
Gryseels *et al*., [36], Cambodia	2 (CD)	2 (FI)	0	4
Jian-Wei *et al.* [43], Myanmar	2 (BD)	4 (FGHI)	0	6
Joshi and Banjara [37] , Nepal	1 (B)	3 (GHI)	0	4
MacFarlane *et al*. [48], PNG	2 (CD)	4 (FGHI)	1 (L)	7
Nonaka *et al*. [38], Lao PDR	4 (BCDE)	3 (GHI)	0	7
Ohnmar *et al*. [49], Myanmar	2 (DE)	4 (FGHI)	0	6
Kyawt-Kyawt-Swe and Pearson, [39], Myanmar	1 (B)	4 (FGHI)	0	5
Sanjana *et al*. [41], Indonesia	3 (BCD)	4 (FGHI)	0	7
Shirayama *et al*. [50], Lao PDR	1 (D)	4 (FGHI)	0	5
Tangjang *et al*. [51], India	0	1 (I)	1 (K)	2
Wangroongsarb *et al*. [40], Thailand	2 (BD)	3 (GHI)	0	5

*Codes refer to Table 2.

## Results

All 28 papers reviewed were subject to careful reading, interpretation and appraisal using a comprehensive critical review approach [34]*.* The review process identified four main themes relating to TM/TH use for malaria in low- to middle-income countries in the Asia-Pacific region: ‘prevalence of TM/TH use’, ‘profile of TM/TH users’, ‘types and timing of TM/TH use’, and ‘reasons for TM/TH use’. Each is outlined in turn below.

### Prevalence of TM/TH use

Eighteen of the 19 papers included in the review reporting quantitative research provide prevalence rates for TM/TH use with regard to malaria. These papers report a wide range of prevalence rates for TM/TH use among people with malaria or among people living in malaria-endemic areas (see Additional file 1). For example, a large-scale survey in endemic areas of northeast India revealed that 39.2% of adults living in a household which had contained a malaria sufferer in the previous three months visited a TH (*Vaidya*) [35]. Another study in northeast Cambodia found 14.4, 37.3 and 40.1% people used herbal treatment, animal sacrifice and coin massage, respectively, to treat malaria [36]. Regardless of the variability in TM and TH user rates across the different studies reviewed, the empirical literature does appear to demonstrate substantial prevalence rates for TM/TH use among malaria patients or people in malaria-endemic areas across a number of Asia-Pacific countries.

The percentage averages of user rates for TM/TH with regard to malaria slightly differ between the large sample studies (n >500) and small sample studies (n <500) in the review. The average prevalence rate for TM/TH use among the larger sample studies was 17.2% while among smaller sample studies it was 13.3%. The prevalence of TM/TH use for malaria treatment in the large and small sample studies ranged from 1 to 40.1% [35-42], and from 1.3 to 32.1%, respectively [43-52].

### Profile of TM/TH users

Sixteen of the articles reviewed provided descriptions of the socio-demographic characteristics of respondents but most failed to provide analyses on the correlation between socio-demographic factors and the use of TM/TH (instead reporting characteristics of wider populations under study). The three exceptions were studies conducted in India, Malaysia and the Philippines, which identified those utilizing TM/TH for malaria as more likely to be: women [45], people with lower educational attainment [35,52], people with lower household income [35], those with farming occupations [35], and, those from ethnic minorities [35]. However, it should be noted that these findings should be interpreted with some caution as are identified from only three studies in the review. The use of traditional herbs was also more popular among women, with men tending to purchase conventional medicine [45]. Meanwhile, there was no gender difference identified regarding the choice to seek treatment across various health providers (TH, governmental and private health services, and self-medication) for malaria in a study in northeast India [35]. No significant correlation between educational background and the use of TM for malaria treatment was identified in a study conducted in the Philippines [45].

Most of the studies conducted in rural or remote areas indicated higher prevalence of TM/TH use (2.5-40.1%) [35-39,41,43-45,48-55] than those conducted in mixed areas of urban, semi-urban, rural, and remote areas (1–4.5%) [40,42,47]. In addition, two papers reporting data from one study in Malaysia found that the prevalence rate of TM/TH use amongst forest-aboriginal people living in more remote areas (41%) was higher than those respondents living in rural area (15.4%) [44,52]. Moreover, two studies in India [35,47] reported that a number of communities living beyond 5 km from the nearest health centre facility were likely to delay seeking treatment from conventional health services and to use TM/TH instead.

### Types of TM/TH use

There are a number of types of TM/TH use for malaria in the articles reviewed and the most popular types of TM employed for malaria treatment or prevention were herbal medicines [38,40,41,43-45,51,52,56]. A Malaysian study reported the use of medicinal plants for curative purposes regarding malaria through their application via bathing or compressing the enlarged spleen during traditional rituals [44], and an Indian qualitative study reported herb use and application [57]. There is evidence of other traditional ways with which Asian Pacific populations attempt to treat or prevent malaria, such as using faith healing [38,50,56], coin massage [36] and massage [58].

### Timing of TM/TH use

The reviewed literature indicates that TM/TH is used for malaria treatment [35-53,55,58-61] and prevention [41,44,50,52,59]. Differences in health-seeking behaviour based upon the temporal aspects of treatment (initial, secondary and/or final treatment) can also be identified from the literature [35,38,43,53,55,56]. The majority of adult participants in India [56], Myanmar [43] and Vanuatu [55] reported that immediate care was not sought and they delayed seeking treatment from a health centre or conventional health provider while initially practicing home remedies followed by TH use. In contrast, prompt seeking of care and treatment for malaria from a health centre or hospital was more common with regard to sick infants and young children [56], children aged under 15 years [43], elderly or young persons with severe symptoms, and for pregnant women [55]. However, one study that interviewed parents or caregivers of children with severe malaria revealed delays in seeking hospital care [42].

Analyses of four studies [35,38,42,53] examining the sequence of use of malaria treatment providers, including government health services and TH, revealed differences in results. Three of these four studies identified different options for treating malaria during initial, secondary and or final treatment. One cross-sectional study conducted in Papua New Guinea (PNG) reported consistent treatment choice (health centre) by respondents across initial as well as second stage of malaria treatment [53]. In contrast to this PNG-based study, research undertaken in India [53] suggests adults living in malaria-endemic areas are more inclined to use a TH (*Vidya*/herbalists) as an initial treatment choice as opposed to a conventional health centre. However, following this initial treatment response, the study participants then utilized government health services for their final treatment and, interestingly, no adults surveyed in this Indian study reported the use of TH as a final treatment choice for malaria [53]. Meanwhile, a study in Lao PDR showed that people who sought initial hospital care were less likely to seek secondary treatment than people who sought TM (faith healing) for initial care. The same study also reported those people who initially visited a conventional health centre had a higher chance of retaining a connection to the health centre for secondary care while people who consulted a TH for their initial treatment were found to be less inclined to also seek a TH for their secondary care [38].

In four of the reviewed studies [52,53,61,62], malaria patients or their carers were reported to use TM/TH in conjunction with biomedical treatments for malaria. For example, a qualitative study in PNG revealed the concurrent use of TM and conventional medicine amongst malaria patients [53] and another study in India reported the concurrent consumption of anti-malarial tablets and consultation with the *gunia* (faith healer) [56]. The respondents in this Indian study described visiting a faith healer as a necessary cultural norm regardless of the treatment options available [56]. Another qualitative study in Indonesia discovered a mixture of concurrent use of TM (specially treated water with prayer, herbal drinks, massage) and conventional health care practices (tablets and injection) for treating malaria in the community [58].

### Reasons for TM/TH use

There is evidence reported in a selection of the papers reviewed that a small number of respondents prefer TM/TH for malaria rather than conventional health services. The most common reasons for TM/TH use for malaria as identified in the reviewed literature are a lack of accessibility to either conventional health services [53,56,57,63] or village health workers [45]. It is important to note that the issue of accessibility is characterized via various features (e.g., geographical convenience and financial accessibility) across the relevant studies in the review. For example, a qualitative study in India [57] revealed that the common use of a TH (*dishari*) related to the perception of a lack of availability of good conventional health services in the locale, the distance to the closest good health facility, and the accessibility of transportation to such a facility. In addition, respondents in a Cambodian study reported the use of TM for malaria as due to conventional medicine being perceived as unaffordable [63].

Findings from the reviewed literature suggest that faith in traditional treatment [55,56,58], and the perception of lower severity of malaria symptoms [53,55,57,63] are amongst the main reasons for seeking TM/TH for malaria treatment and prevention. For example, a study in PNG [53] revealed that participants would seek help from a TH if they believed only a TH was able to cure the disease. Similar findings were demonstrated in a study in India [56] which found belief in traditional healing motivated people to utilize a TH after engaging home remedies.

### Appraisal outcomes

A quality appraisal scoring system was employed to evaluate the 19 papers included in the review that reported quantitative research. Although the majority (11) of the 19 articles report the use of a rigorous study design (Table 3), there also appears to be a number of methodological limitations to the existing literature regarding TM/TH use for malaria in low- to middle-income countries in the Asia Pacific region. Only eight of the 19 articles reporting quantitative research utilized a sample size of more than 500 respondents (ranging from 700 to 1,989 participants) and 11 articles described samples of less than 500 respondents (ranging from 99 to 446 participants). One Thailand-based study surveyed 1,719 participants and covered large geographical areas of the country [40]. Nevertheless, none of the studies reviewed reported on national-scale datasets or a nationally representative sample, the majority of study samples involved only one to two districts/regions [38,41,44-46,49,50,52] and none examined large samples across the remote-rural–urban spectrum. Almost all of the studies were conducted in rural and remote areas [35,37-39,41,43-45,48-54] and only three studies were administered in both urban or semi-urban *and* rural or remote areas [40,42,56]. Moreover, two studies only targeted specific sub-populations, such as Nasioi ethnic people in PNG [48] and Myanmar and Cambodian migrants in Thailand border areas [40].

There are some methodological constraints found in the reviewed papers regarding non-response bias, recall bias, confirmation of the status of malaria patients as well as the extent to which studies provided a well-articulated definition for TM/TH for respondents, and/or provided an opportunity for respondents to name the type of TM/TH used. Nine of the 19 papers reporting quantitative studies outlined a response rate (with all nine achieving a 75% response rate or higher). Most studies addressed the accuracy of recall (previous two weeks to one year) yet four papers did not provide any information on the methodology which may influence recall [36,37,46,51] and one study appears to have been subject to possible significant recall bias (a two-year recall period) [39]. Although providing evidence of the presence of malaria parasites in human blood status is burdensome in rural and remote areas in low- to middle-income countries, five studies reported the use of a parasitological method for confirming malaria patient status [38,42,45,46,49] with the rest of the studies evaluating malaria status of the respondents based on self-reported or physician-reported clinical conditions [35-37,39-41,43,44,47,48,50-53]. Importantly, only one quantitative study examined the perceived effectiveness of the use of TM/TH for malaria amongst the community [49].

No study included in the review provided a clear definition of TM/TH to the respondents, and only four studies allowed respondents to name the TM/TH type using open-ended questionnaire design [44,45,51,52]. There does appear to be ambiguity across the literature when evaluating TM/TH use regarding malaria. None of the studies focused in-depth upon the prevalence and characteristic of TM/TH user for malaria. Most research to date has examined conventional health centre/malaria programme utilization for malaria treatment and prevention, including only a consideration of TM/TH use as one (often peripheral) issue amongst a large number of competing foci and as a consequence the data regarding TM/TH use for malaria remain limited.

## Discussion

This paper provides the first critical, comprehensive review of the evidence base of TM/TH use among malaria patients as well as among people in malaria-endemic areas in low- to middle-income countries in the Asia-Pacific region. The studies included in the review were conducted in 12 countries and eight of 28 papers reported research undertaken in India. Thirteen of the 28 articles covered in this paper were published over the past five years revealing an intensification of research focusing on malaria treatment-seeking behaviour and practice of malaria treatment and/or prevention over recent years in low- to middle-income countries across the Asia Pacific region. Despite this emerging focus upon malaria treatment-seeking behaviour, the review has identified several gaps in the scientific literature relating to TM/TH use.

There is a general lack of research focus on TM/TH utilization for malaria. Only one study focused *exclusively* on the use of TM for malaria [50]. A better understanding of health-seeking behaviour of people in malaria-endemic areas, especially amongst those suffering from fever related to malaria, is important for effective illness management and control. Although the most common provider chosen by most participants in the studies reviewed are the services found at conventional health centres, the prevalence rate of TM/TH use identified demonstrates a significant number of people using TM/TH for malaria as to warrant further in-depth attention. It is also important to note that the correlation between socio-demographic factors and the use of TM/TH was not often mentioned in the papers reviewed. This constitutes a significant gap in our understanding of TM/TH use for malaria. More detailed information regarding who is likely to utilize TM/TH for malaria and under what circumstances will help in the planning and deliver of malaria health education and public health campaigns.

This review shows concurrent use of TM/TH with conventional medicine is common in a number of communities. Concurrent TM/TH use will likely place patients at risk of possible adverse interactions [63] that may lead to minor adverse effects or even possibly death [64,65]. The possibility of herbal medicine and anti-malarial drug interactions has been noted [66]. The concurrent use of TM/TH with conventional medicines for malaria also warrants further investigation with specific focus upon the decision-making process and the communication between health staff and patient and among health staff and TM providers [67,68]. It is also important that future research identify and examine the specific types of herbal medicines used by malaria patients with a view to helping ensure effective and safe care. Unfortunately no paper in the review examined the scientific pharmacological details of the herbal medicines used and there is need for further research to examine the relationship between the TM used and their pharmacological properties and evidence-base.

The qualitative research identified in the review shows a belief in the benefit of TH motivates individuals to either visit a TH instead of using the services of a conventional health centre or to use TM/TH concurrent to conventional health centre service use. It is vital malaria research consider local cultural contexts (i.e. the behaviours, perceptions and beliefs of the local community which often closely relate to TM/TH use) in order to help ensure conventional programs and interventions designed to manage and/or treat malaria attract optimal community acceptance..

None of the articles reviewed provides national data coverage and large-scale national surveys of TM/TH use for malaria are required. Nationally representative research of TM/TH usage for malaria treatment and prevention will allow examination of numerous factors related to TM/TH consumption [69] as well as provide considerable statistical power for answering important research questions.

The ability to generalize from or compare findings across contemporary studies remains challenging due to the variations in research design, the absence of TM/TH definitions employed between studies and the fact that the majority of articles identified report studies which have been conducted in a fairly select group of countries. There is a need for further research on this substantive topic to be conducted in a wider range of settings in order to provide coverage and representation across the full Asia-Pacific region.

It is important to note the limitations of this critical review. The papers reviewed were restricted to English-only publications and the omission of non-English literature may introduce some bias. Nevertheless, the review reports the first critical, systematic evaluation of the research literature on TM/TH use for malaria and in malaria-endemic areas in the Asia-Pacific region providing a useful resource for practitioners, policymakers and researchers interested in understanding and addressing the challenges of managing and treating malaria in the Asia-Pacific region.

## Conclusion

This paper reports the first critical overview and reveals crucial insights into the prevalence and profile of TM/TH use among malaria patients and among people in malaria-endemic areas with implications for TM/TH users, practitioners and health policy makers. All managing and providing conventional programmes, treatment and care with regard to malaria in the Asia-Pacific region should remain mindful of the possible use of TM/TH amongst community members and patients. Further research on this important issue is required in order to fully inform all stakeholders engaged in preventing and treating malaria amongst Asia-Pacific communities in low to middle income countries.

## Additional files

Additional file 1:
**Quantitative studies on TM/TH use for Malaria in low to middle income countries in the Asia Pacific region, January 2003- October 2014.**
Additional file 2:
**Qualitative studies on TM/TH use for Malaria in low to middle income countries in the Asia Pacific region, January 2003- October 2014.**
Additional file 3:
**Quality scoring of each article reviewed (Quantitative Studies).**

## Abbreviations

PNG: Papua New Guinea
TH: Traditional healer
TM: Traditional medicine
