# Supplementary material for: A critical review of traditional medicine and traditional healer use for malaria and among people in malaria-endemic areas: contemporary research in low to middle-income Asia-Pacific countries
Source: Malar J. 2015 Mar 1;14:98. doi: 10.1186/s12936-015-0593-7 (PMC4350610; doi:10.1186/s12936-015-0593-7)
Supplement: Additional file 1: — Quantitative studies on TM/TH use for Malaria in low to middle income countries in the Asia Pacific region, January 2003- October 2014. [file 12936_2015_593_MOESM1_ESM.pdf]

Additional File 2. Quantitative studies on TM/TH use for Malaria in low to middle income countries in the Asia Pacific region, January 2003- October 2014

Author/ Year	Country	Areas	Design	Sampling technique	Study population	Samples (n)	Use rate of traditional medicine/ healers/ therapies		Type of Popular Medicine or Therapies	
							**Prevention**	**Treatment**	**Prevention**	**Treatment**
Al-Adhroey, (2010b)	Malaysia	Forest-aboriginal and rural	A cross-sectional survey	Universal sampling	adult households who had or had not suffered a malaria crisis	223	Medicinal plants (21.0%)<br>Beliefs in witchcraft and sorcery (5.8%)	Medicinal plants (7.1%)	Mosquito bed nets (61.8%)	Health Centre (95.1%)
Al-Adhroey (2010)	Malaysia	Forest-aboriginal and rural	A cross-sectional ethno-botanical survey	- (mentioned in Al-Adhroey 2010b)	Adult aboriginal household' members, out patients of clinic in rural areas, and traditional healers	n=233<br>n = 10 (traditional healers)	-	Antimalarial plants 21.0% (47/233)	-	Antimalarial plants
Al-Taiar (2009)	Yemen	Urban, semi-urban and rural	FGDs and questionnaire based interviews among	Systematic random sampling for recruiting parents of non-malaria patients	Parents or guardians either at the referral hospital (severe malaria patients), health centre (mild malaria patients) or at home (non- malaria patients)	1113	-	Traditional healers 1%	-	**Initial step:** Antipyretic/ sponging (57%)<br>**Second step:** Clinic (50%)
Bell (2005)	Philippines	Remote areas	A parasitemic survey and interviews	Systematic sampling	Village Health Workers (VHWs) and adult household members	n=271 (parasitemic survey)<br>n=72 (adult household members)<br>n=27 VHWs	-	**Initial step**: Herbal medicine (22%)<br>**Second step**: Herbal medicine (8.3%)	-	**Initial step:** Health service (56%)<br>**Second step:** Health service (69.4%)
Borah (2004)	India	Rural	-	-	Acute febrile patients during malaria outbreaks	184	-	Traditional practitioners (3.8%)	-	Public health facility (60.3%)
Chaturvedi (2009)	India	Rural and remote areas	Household survey	Two stage sampling	Adult household with household member that were suffered from malaria previously	1,989	-	Traditional healer *(Vaidya/herbalist)* (39.2%)	-	**Initial choice**: traditional healer *(Vaidya/herbalist) :* (39.2%) Government health service 29.3%<br>**Final choice**: Government Health Service 65.5%

Author/ Year	Country	Areas	Design	Sampling technique	Study population	Samples (n)	Use rate of traditional medicine/ healers/ therapies		Type of Popular Medicine or Therapies	
							**Prevention**	**Treatment**	**Prevention**	**Treatment**
Das & Ravindran (2010)	India	Rural areas	A cross-sectional community-based survey among	Multi-stage sampling	Adults (non-pregnant women) who had fever with chills or their care takers in the last two weeks and interview of providers.	300 patients<br>23 malaria providers	-	Traditional healers 0.3%<br>Homeopathy 1%<br>Total Traditional Medicine: 4/300 = 1.33%	-	Government health centre 35.7%<br>Less qualified providers 32.3%
Davy (2010)	PNG	Rural Areas	A cross sectional household survey	Sampling was based on the average time to walk to the nearest health centre	Individuals who diagnosed for malaria or reported fever and or convulsions (without asking chills).	928 household members	-	-	-	**Initial choice:** -<br>**Second treatment:** -<br>No user rates but provide Confidence Interval.
Gryseels (2013)	Cambodia	Rural and remote areas	Mixed method study. A qualitative ethnographic study (participant observation and interview) and a quantitative study (two household surveys)	Purposive sampling method for qualitative study<br>Random sampling from 113 chosen villages for quantitative study	Household members in indigenous ethnic areas	126 recorded interviews and 32 additional unrecorded interviews<br><br>824 respondents<br>711 respondents for cross sectional survey	-	Combined with other treatments:<br>Animal sacrifice 37.3% (265/711)<br>Herbal treatment 14.5% (103/711)<br>Coin massage 40.1% (285/711)<br><br>Only animal sacrifice, herbal treatment and coin massage 0.1% (1/711)	-	Combination of modern (injections, infusion, pills) and traditional treatments (animal sacrifice, herbal treatment and coin massage) (77.9%)
Jian-Wei (2012)	Myanmar	Rural/remote areas	A cross sectional questionnaire-based household survey among household heads with a patient that has symptoms of malaria and in-depth interviews to key informants.	convenient sampling	Household members with presumptive malaria patients	369 households with presumptive malaria patients,<br><br>36 key informants	-	13% (42/323) who sought treatment) took herbs<br>7.3% (27/323) sought advice or treatment from other sources such as a traditional healer, a friend or relative	-	Retail sector (drug peddlers, shops and market stalls):79.6% (257/323)

Author/ Year	Country	Areas	Design	Sampling technique	Study population	Samples (n)	Use rate of traditional medicine/ healers/ therapies		Type of Popular Medicine or Therapies	
							**Prevention**	**Treatment**	**Prevention**	**Treatment**
Joshi & Banjara (2008)	Nepal	Rural areas	A cross-sectional study using survey and FGDs	Multi-stage sampling	Community in malaria endemic areas	1330 respondents	-	Traditional healers (16.1%)	-	Most of respondents consulted with the health facilities (no percentage)
Macfarlane & Alpers (2009)	PNG	Rural areas	A non-experimental, cross-sectional study design and focused ethnographic approach	Convenience sampling	Specific ethnic, the Nasioi people	200 community members	-	Traditional medicine 32.1% (62/193)	-	-
Nonaka (2009)	Lao PDR	Rural and remote areas	A survey using a pre-tested, structured-questionnaire and blood test for malaria among the community members	Purposive sampling	Head/member of household in malaria endemic areas	745 household heads/ members (survey)<br><br>3624 community members (blood test)	-	**Initial Care:**<br>Faith healing 15.1% (94/624)<br>Herbal remedy 1.9% (12/624)<br>**Secondary care:**<br>Faith healing 28.2% (72/255)<br>Herbal remedy 3.1% (8/255)	-	**Initial Care:**<br>Hospitals 23.7 % (148/624)<br>Health centres 23.7% (148/624)<br>**Secondary care:**<br>Faith healing 28.2% (72/255)<br>Health centre 25.1% (64/255)
Ohnmar (2010)	Myanmar	Remote areas	A cross-sectional house-to-house survey among community	Cluster sampling	Household heads/ members	446	-	Traditional Medicine 7% (31/446)<br>168 (37.7%) both TM and modern medicine<br>164 (67.5%) people used TM for self-care.	-	Traditional medicine
Pearson (2004)	Myanmar	Rural and remote areas	A survey and FGD among community members (mixed	Two-stage sampling	Household heads/ members	700	-	Traditional practitioner 3% (17/700)<br>Herbal remedies 0.2% (1/700)	-	Self-medication using unspecified drugs 42.1% (238)

Author/ Year	Country	Areas	Design	Sampling technique	Study population	Samples (n)	Use rate of traditional medicine/ healers/ therapies		Type of Popular Medicine or Therapies	
							Prevention	Treatment	Prevention	Treatment
Sanjana (2006)	Indonesia	Rural areas	A survey assessed malaria knowledge, attitudes, and practices in communities experiencing epidemic malaria	Two-stage sampling	Household heads/ members	1000 households adults	-	Prevention Traditional medicine/jamu 18.4% No respondent using traditional medicine for Treatment	-	Health centre 29.8% of 409 malaria patients
Shirayama (2006)	Lao PDR	Rural areas	A community-based cross-sectional survey	Purposive sampling	Household heads/ members	240 household heads/adults	Bum trees or herbs 3.3% (8/240)	Traditional medicine or religious ceremony 2.5% (6/240)	Bed net 92.9% (223/240)	over- counter drugs (self - treatment) 52.9% (127/240)
Tangjang (2010)	India	Remote areas	A house-to-house surveys among the ethnic community	Random sampling	The ethnic community members	237 informants (including 84 traditional plant practitioners) Rural	-	Medicinal plants 7.6% (18/237)	-	Medicinal plants (*Coptis teeta)*
Wangroon gsarb (2011)	Thailand	Rural and urban areas	A cross-sectional and household survey methods	Respondent-driven sampling	Migrants in Thailand-Cambodia border	1800 participants	-	Herbal medicine 4.5%	-	Herbal medicine 4.5%

Note: FGDs= Focus Group Discussions, VHW=Village Health Worker, PNG=Papua New Guinea
