# Supplementary material for: A critical review of traditional medicine and traditional healer use for malaria and among people in malaria-endemic areas: contemporary research in low to middle-income Asia-Pacific countries
Source: Malar J. 2015 Mar 1;14:98. doi: 10.1186/s12936-015-0593-7 (PMC4350610; doi:10.1186/s12936-015-0593-7)
Supplement: Additional file 2: — Qualitative studies on TM/TH use for Malaria in low to middle income countries in the Asia Pacific region, January 2003- October 2014. [file 12936_2015_593_MOESM2_ESM.pdf]

Additional File 3. Qualitative studies on TM/TH use for Malaria in low to middle income countries in the Asia Pacific region, January 2003- October 2014

Author/Year/ Country	Design/Sampling	Samples (n) & Areas	Use Rate of Traditional Medicine/Therapies/ healers Used	Type of Popular Medicine or Therapies Used/Behaviour	Reason for Use	Special Remark
Das et al., 2013 India	26 community FGD and 40 interviews<br>Purposive sampling	272 respondents<br>Two districts<br>Rural areas	NA	Home remedies/herbs<br>Herbalist<br>Faith healers	○ low cost<br>○ easy availability<br>○ community's faith on traditional methods of healing	Few days delay of seeking health care and engaging in home remedies until the disease worsen, except for sick infants and young children
Oconnell et al., 2013 Cambodia	In-depth interviews (IDIs and focus group discussions (FGDs)<br>A non-probability, purposive sample and snowball sampling method	16 Interviews<br>12 FGD<br><br>Some districts in two provinces	NA	Majority used medication (pre-package drugs)<br>Some used Traditional home remedies	NA	
Sabin et al., 2010 India	IDIs and FGDs	32 in-depth interviews<br>6 FGDs<br>Total 73 pregnant women<br>One district hospital catchment area.<br>Semi-urban and rural areas.	Traditional prevention approaches (69% informants from 32 in-depth interviews and 5 out of 6 FGDs)<br>Traditional remedy for treatment (5 out of 24 informants)<br>2 out 5 used TM concurrently with conventional medicine	63% see the doctor or nurse for malaria treatment during pregnancy<br><br>69% use traditional method for malaria prevention	○ Effectiveness<br>○ Cost<br>○ Availability<br>○ Accessibility	
Shah et al., 2014 Pakistan	Semi structured interviews and questionnaires for ethno-botanical survey.	55 informants.<br>Mostly remote areas.	60% of the respondents prefer herbal treatment by herbalist (hakim) or self-treatment with locally available medicinal plants.	Herbal medicine	○ More economical<br>○ Reliable<br>○ Less side effect	The knowledgeable users of traditional medicine are older men and women, nomads, shepherds and local healers.
Sundararajan et al., 2013 India	Interviews and FGDs.<br><br>Random and purposive sampling for selecting villages and participants.	84 informants: 76 participated in FGDs and 8 informants provided individual interview.	NA	Traditional healers	○ Effectiveness to symptoms relief<br>○ Cost<br>○ Perceived side effects	Traditional healers were sought first and follow recommendation for referral to an allopath if rituals do not alleviate symptoms.<br>Although villagers and pujaris (traditional healers) recognize they cannot treat malaria.
Tynan et al., 2011 Vanuatu	FGDs, participatory workshops and key informant interviews (KIIs), as well as structured and unstructured observation and field notes	9 FGD<br>12 KII<br>7 participatory workshop	NA	The *kastom* (traditional) medicine or other home remedies (a typical first treatment resort of any type of fever, including malaria fever)	○ Positive and negative previous experience with medical treatment<br>○ various geographical (accessibility/remote) and ideological reasons (belief to	It was routinely claimed that if the kastom medicine or home remedies did not work, respondents would then consider taking the person or themselves to the aid post for further investigation or seeking other forms of biomedical intervention.<br>Children and pregnant women will seek health centre care.

Author/Year/ Country	Design/Sampling	Samples (n) & Areas	Use Rate of Traditional Medicine/Therapies/ healers Used	Type of Popular Medicine or Therapies Used/Behaviour	Reason for Use	Special Remark
					traditional healers/medicine)	
Utarini et al., 2003 Indonesia	An applied qualitative method, Rapid Assessment Procedures among rural community<br><br>snow-ball technique	66 respondents<br>38 free-listings,<br>28 in-depth interviews,<br>seven FGDs and unstructured observation	NA	Drinking herbs<br>Massage<br>Traditional healer	○ Cost considerations<br>○ more common in areas with limited access to over-the-counter drugs,<br>○ when modern medicine does not work	The health center was used but if not deemed useful most people would shift back to traditional medicine/healer services due to cost considerations
Utarini et al., 2007 Indonesia	A one-year longitudinal observational study among community in a convenience sample Purposive sampling	24 interactions selected over a one-year period and representing different Village Malaria Workers, clinical/confirmed malaria cases, and men/women endemic areas	NA	self-medication	Perceived treatment effectiveness	Traditional healers and herbal medicines were rarely used, while self-medication (“drug only”) was the third most common action taken until three days post-symptom recognition.
Vijayakumar et al., 2009 India	KIIs and FGDs<br>A multistage stratified random sampling method	36 KIIs<br>24 FGDs	21% making smoke from bone	Treatment:<br>Traditional healers<br>Herbs<br>Prevention:<br>making bone fire (for smoke)	Accessibility (distance)<br>malaria fever can be cured with herbal medicines	
