# Supplementary material for: A critical review of traditional medicine and traditional healer use for malaria and among people in malaria-endemic areas: contemporary research in low to middle-income Asia-Pacific countries
Source: Malar J. 2015 Mar 1;14:98. doi: 10.1186/s12936-015-0593-7 (PMC4350610; doi:10.1186/s12936-015-0593-7)
Supplement: Additional file 3: — Quality scoring of each article reviewed (Quantitative Studies). [file 12936_2015_593_MOESM3_ESM.pdf]

Additional File 4: Quality scoring of each article reviewed (Quantitative Studies)

Author/Year /Country	Methodology					Sub Score	Reporting of Participants'Characteristic				Sub Score				Sub Score	Total Score
	**National Represen-tative**	**n>500**	**Response Rate >75%**	**No Recall Bias (within 12 month)**	**Confirmed Malaria Patients**		**Status of Malaria**	**Age**	**Socio-economic Indicators**	**Type of Location (Urban/Rural/ Remote)**		**Provided Definition of TM/ Therapies**	**TM Naming (open questions)**	**Assessed Use of TM/ Therapies**		
	**A**	**B**	**C**	**D**	**E**		**F**	**G**	**H**	**I**		**J**	**K**	**L**		
Al-Adhroey et al, 2010b, Malaysia	X<br><br>One district<br>Universal sampling	X<br><br>223	√<br><br>85.7%	√<br><br>Last year	NA	**2**	√	√	√<br><br>Education<br>Race<br>Sex<br>Religion<br>Gender	√<br><br>Rural & forest areas	**4**	NA<br><br>KAP malaria treatment prevention	V<br><br>Medicinal plants<br>Herbal medicine<br>Witchcraft	X	**1**	**7**
Al-Adhroey et al, 2010, ethno Malaysia	X<br><br>One district<br>Universal sampling	X<br><br>233	NA	√<br><br>Last year	NA	**1**	√	NA	NA	√<br><br>Rural & forest areas	**2**	NA<br><br>Ethno-botanologi cal survey	V<br><br>Focus on names of medicinal plants	X	**1**	**4**
Al-Taiar et al, 2009 Yemen	X<br><br>One province<br>NA sampling method	√<br><br>1113	NA	√<br><br>Present patient	√<br><br>Parasitologic al analysis	**3**	√	√	√	√<br><br>urban, semi-urban and rural areas	**4**	NA	NA	NA	**0**	**7**<br><br>**Limited info on TM**
Bell et al, 2005 Philippines	X<br><br>Villages<br><br>Systematic Sampling	X<br><br>271 patients<br>27 VHWs<br>72 adult house-holds	√<br><br>100% (patients)<br>77.1% VHWs	√<br><br>In previous 12 months	√<br><br>Parasitologic al analysis	**3**	√	√	√	√<br><br>Remote areas	**4**	NA	V	NA	**1**	**8**
Borah et al 2004 India	X<br><br>One district	X<br><br>184 acute febrile patients/g uardians	NA	NA	√<br><br>microscopica l examina-tion	**1**	√	√	√	X	**3**	NA	NA	NA	**0**	**4**
Chaturvedi et al, 2009 India	X<br>Two districts<br>Two stage sampling (first, probability propotional to size sampling and second systematic sampling)	√<br><br>1,989	√<br><br>99.4%	√<br><br>In the last 3 months	X<br><br>diagnosed by the symptoms and clinical manifestatio ns of the disease	**3**	√	√	√<br><br>Education<br>Race<br>Residential area<br>Gender	√<br><br>Rural and remote areas	**4**	√	NA	X	**1**	**8**

Author/Year /Country	Methodology					Sub Score	Reporting of Participants'Characteristic				Sub Score				Sub Score	Total Score
	**National Represen-tative**	**n>500**	**Response Rate >75%**	**No Recall Bias (within 12 month)**	**Confirmed Malaria Patients**		**Status of Malaria**	**Age**	**Socio-economic Indicators**	**Type of Location (Urban/Rural/ Remote)**		**Provided Definition of TM/ Therapies**	**TM Naming (open questions)**	**Assessed Use of TM/ Therapies**		
Das and Ravindran, 2010<br>India	X<br>One district<br>Multi stage sampling	X<br>281	√<br>100%	√<br>In the last two weeks	X<br>Fever with chills	**2**	√	√	√	√<br>Rural, urban, remote areas	**4**	NA<br>Only definition of less qualified providers	X<br>Close ended questions	X	**0**	**6**
Davy et al 2010<br>PNG	X<br>Two districts Information on sampling technique NA	√<br>928	√<br>98.6%	√<br>In the previous 4 weeks	X<br>Presumpti-ve malaria	**3**	√	√	√	√<br>Rural areas	**4**	NA	NA	NA	**0**	**7**
Gryseels (2013)	X<br>Two villages	√<br>824	√<br>91.5%	NA	NA	**2**	√	NA	NA	√<br>Remote/rural	**2**	NA	NA	NA	**0**	**4**
Xu et al 2012<br>Myanmar	X<br>Two districts Sampling technique NA (only mentioned first household were randomly chosen)	√<br>718 households	NA	√<br>In the previous 2 weeks	X<br>Presumpti-ve malaria	**2**	√	√	√	√<br>Remote areas	**4**	NA	NA	NA	**0**	**6**
Joshi and Banjara 2008<br>Nepal	X<br>Three districts Multistage sampling	√<br>1330 household members	NA	NA	X	**1**	NA	√	√	√<br>Rural areas	**3**	NA	NA	NA	**0**	**4**
MacFarlane et al 2009<br>PNG	X<br>One ethnic areas Convenience sampling	X<br>200 community members	√<br>98.5%	√<br>In the last four weeks	X<br>Presumpti-ve malaria	**2**	√	√	√	√<br>Remote areas	**4**	NA	NA	√	**1**	**7**
Nonaka et al 2009<br>Lao PDR	X<br>One district<br>Technique sampling NA	√<br>745 (Interview) 3264 (blood test)	√<br>86.2% (survey)	In the past year	√<br>13.7% of 3264	**4**	√	√	√	√<br>Rural and remote areas	**4**	NA	NA	NA	**0**	**8**
Ohnmar et al 2010<br>Myanmar	X<br>One district Cluster sampling	X<br>357 key household respondents<br>446 patients	NA	√<br>In the past 3 months	√<br>80%	**2**	√	√	√	√<br>Remote areas	**4**	NA	NA	NA	**0**	**6**

Author/Year /Country	Methodology					Sub Score	Reporting of Participants'Characteristic				Sub Score				Sub Score	Total Score
	**National Represen-**	**n>500**	**Response**	**No Recall**	**Confirmed**		**Status**	**Age**	**Socio-**	**Type of Location**		**Provided**	**TM**	**Assessed**		

	tative		Rate >75%	Bias (within 12 month)	Malaria Patients		of Malaria		economic Indicators	(Urban/Rural/ Remote)		Definition of TM/ Therapies	Naming (open questions)	Use of TM/ Therapies		
Pearson 2004 Myanmar	X<br><br>One township	√<br><br>700 Households adults	NA	X<br><br>Within 2 years	X<br><br>Presumptive malaria	**1**	√	√	√	√<br><br>Rural and remote areas	**4**	NA	NA	NA	**0**	**5**
Sanjana et al 2006 Indonesia	X<br><br>nine sub-districts Random sampling	√<br><br>1000 Households adults	√<br><br>99%	√<br><br>In the past 12 months	X<br><br>Presumptive malaria	**3**	√	√	√	√<br><br>Rural areas	**4**	NA	NA	NA	**0**	**7**
Shirayama et al 2006 Lao PDR	X<br><br>One district	X<br><br>240 households	NA<br><br>Not stated but samples seem to response to all questions	√<br><br>in the past one year	X<br><br>49.2% (118/240)<br><br>Presumpti-ve malaria (laboratory analised if available)	**1**	√	√	√	√<br><br>Rural	**4**	X	X	X	**0**	**5**
Tangjang et al 2010 India	X<br><br>three districts	X<br><br>237 informants	NA	NA	NA	**0**	X	NA	NA	√<br><br>Rural	**1**	X	√	X	**1**	**2**
Wangroongsarb et al 2011 Thailand	X<br><br>3 provinces	√<br><br>1800	NA	√<br><br>(in the previous first month)	NA	**2**	NA	√	√	√<br><br>Rural and urban	3	X	X	**x**	0	**5**
